# A cross-sectional study on sarcopenia using different methods: reference values for healthy Saudi young men

**DOI:** 10.1186/s12891-017-1483-7

**Published:** 2017-03-21

**Authors:** Shaea A. Alkahtani

**Affiliations:** Department of Exercise Physiology, College of Sport Sciences and Physical Activity, King Saud University, PO Box 1949, Riyadh, 11441 Saudi Arabia

**Keywords:** Sarcopenia, Appendicular lean mass, Hand grip strength, Saudi men, Dual-energy X-ray absorptiometry, Bioelectrical impedance analysis

## Abstract

**Background:**

The aim of this study was to determine reference values for sarcopenia indices using different methods in healthy Saudi young men.

**Methods:**

Participants included 232 Saudi men aged between 20 and 35 years. The study measured anthropometric indices, blood pressure, hand grip strength, and lean muscle mass using dual-energy X-ray absorptiometry (DXA), and bioelectrical impedance analysis (BIA) was performed using Inbody 770 and Tanita 980 devices.

**Results:**

Using DXA, the mean value of appendicular lean mass divided by the height squared (ALM/ht^2^) was found to be 8.97 ± 1.23 kg/m^2^; hand grip strength measured 42.8 ± 7.6 kg. While the differences between DXA and BIA (Tanita) were significant for all parameters, the differences between DXA and Inbody values were significant only for ALM parameters. Inbody sensitivity and specificity values were 73% and 95.9%, respectively. The kappa (*P* = 0.80) and *p* values (*P* < 0.001) showed good agreement between Inbody and DXA, whereas Tanita sensitivity and specificity values were 54.2% and 98.3%, respectively. Bland-Altman plots for differences in lean mass values between Tanita, Inbody, and DXA methods showed very high bias for Tanita and DXA, with significant differences (*P* < 0.001).

**Conclusions:**

The cut-off values for sarcopenia indices for Saudi young men are different from those of other ethnicities. The use of tailored cut-off reference values instead of a general cut-off for BIA devices is recommended.

## Background

Sarcopenia has been defined as muscle mass loss, and dynapenia has been defined as muscle strength loss [1]. The Asian Working Group for Sarcopenia (AWGS) defined sarcopenia as low muscle mass plus low muscle strength and/or low physical performance [2]. The loss of muscle mass begins at 30 years of age, and loss is the greatest after 50 years of age [3]; muscle strength reaches its maximal level at 30 years of age, and is sustained until approximately 50 years, when it begins to decline [4]. The prevalence of sarcopenia increases from the third to sixth decades, and remains relatively constant thereafter [5]. Although sarcopenia is partially a geriatric syndrome, it is to a great extent a reversible phenomenon. For example, there is substantial inter-individual variability of up to 40% in the loss of muscle mass and muscle strength among older individuals, due to genetics and lifestyle.

Sarcopenia can be estimated using a skeletal mass index (SMI), calculated as muscle mass/body mass × 100. The mean SMI value among young American men aged between 18 and 39 was 42.5 ± 5.5%, and above 37% was considered normal; SMI between 31.5 and 37% was considered sarcopenia class I and SMI less than 31.5% was considered class II [5]. Sarcopenia can also be estimated using appendicular lean mass (ALM) and/or ALM/ht^2^. Using three different indices of lean mass, ALM yielded the best results, when compared with total lean mass (TLM)/ht^2^ and SMI in the diagnosis of low muscle mass [6]. The AWGS agreed on assigning values using two standard deviations below the mean ALM for young adults or the lower quintile for an older group. The AWGS definition of sarcopenia suggested that diagnosis could be initiated with the measurement of hand grip strength, followed by the measurement of gait speed when hand grip strength decreased, and lastly by the measurement of muscle mass when hand grip strength and gait speed were both decreased. They suggested the use of the following cut-off criteria: ALM of 7.0 kg/m^2^ for men and 5.4 kg/m^2^ for women using DXA, ALM of 7.0 kg/m^2^ for men and 5.7 kg/m^2^ for women using BIA, hand grip strength <26 kg for men and <18 kg for women, and gait speed <0.8 m/s [2]. Different methods of hand grip strength measures have been reported, using dominant or both hands, considering that dominant hand is stronger than other hand by 10% for right handed people, whereas its force is similar among left handed people, and reliability of one trial is similar to three trial particularly among untrained populations, and hand grip force could reduce during repeated trials [7]. Some studies take the mean of measures [8], but the majority of studies take maximal reading of dominant hand [9].

Different methods such as magnetic resonance imaging (MRI), computed tomography (CT), and DXA can be used to accurately estimate muscle mass. While MRI and CT are optimal for estimating muscle mass, DXA is preferable for clinical and research use. BIA is another method that has been used for decades, and the European Working Group on Sarcopenia in Older People (EWGSOP) has considered BIA a good portable alternative to DXA [1]. However, different brands and models of BIA devices have been used to measure muscle mass and predict sarcopenia, including bioelectrical impedance spectroscopy models [10], Xitron Technologies [11], Valhalla Bio-Resistance Analyzer [5], Tanita BC [12], and Inbody Biospace [13]. Although these BIA types use the same technique by sending a current through the body, different BIA types use different frequencies and resistance levels. Most studies examined the validity of BIA products by using DXA, but there is a need to examine the validity of different BIA types using DXA in the same cohort in order to evaluate the differences between these devices in the estimation of muscle mass.

The EWGSOP recommended using reference values for a population based on the values for healthy young adults, rather than using predictive values for a reference population. Ethnicity is associated with the magnitude of muscle mass, and affects the degree of decline of skeletal muscle mass [14]. In addition, the estimation of skeletal muscle mass using BIA is influenced by racial differences; skeletal muscle mass is underestimated for Asians when using reference equations for Caucasians, although the equations are applicable for Hispanics and African Americans [15]. The AWGS was established in 2013, and only includes countries from East Asia (China, Hong Kong, Japan, Korea, Malaysia, Taiwan, and Thailand). This working group hopes to promote sarcopenia research in Asia [13]. Data from the South and West of Asia are needed, particularly because the ethnicities in these countries differ from those of East Asia. The current study aimed to determine reference value for sarcopenia indices among young Saudi men, using DXA and different BIA devices.

## Methods

### Participant characteristics

The participants included 232 apparently healthy men aged between 20 and 35 years old. Participants were recruited via notice board at King Khaled Hospital at King Saud University (KSU) and through social media, with no specific criteria listed; thus, the sample size included a wide range of the general population, including obese and athletic individuals. All voluntary participants who expressed their interest in the study signed a consent form prior to participation.

### Study design

The current cross-sectional study was performed at the Laboratory of Body Composition in the Department of Exercise Physiology, College of Sport Sciences and Physical Activity, KSU.

### Data management

The study measured anthropometric indices, hand grip strength, knee extensors strength, and body composition using dual-energy X-ray absorptiometry (DXA) and bioelectrical impedance analysis (BIA).

#### Anthropometry

Height was measured to the nearest 0.5 cm, and weight was measured to the nearest 0.1 kg, using a digital stand scale. Waist circumference was measured to the nearest 0.1 cm at the umbilicus using measuring tape. Participants were instructed to exhale while standing, and a research assistant took two waist measurements.

#### Body composition

##### Dual-energy X-ray absorptiometry (DXA)

The Body Composition Laboratory in the Department of Exercise Physiology, College of Sport Sciences and Physical Activity, KSU, has access to a Lunar iDXA General Electric machine (Lunar iDXA, GE Healthcare, USA). Prior to each day’s tests, Quality Assurance Calibration was performed automatically using a block that contains bone equivalents of known width and density; the system should confirm that the test is passed.

All participants confirmed that they had no radiation exposure such as X-rays in the prior two weeks, and had no frequent exposure to radiation in the prior year. Participants were informed of the procedure in advance. Participants’ data were inserted, and ethnicity was determined as white as recommended by the operator, such that approximately five participants who are originally from Africa were determined as black and there data were excluded. Participants were fitted on a supine position on the bed, and Velcro straps were used for ankles and knees when needed during the scan. Participants were required to remain motionless while the arm of the machine passes over their body, which takes approximately 6 min for average adults. Output was immediately printed at the end of test.

##### Bioelectrical impedance analysis (BIA)

Inbody 770 (Inbody Co., LTD, Seoul, Korea) and Tanita MC-980MA (Tanita Corporation, Tokyo, Japan) BIA devices were used to measure body composition. BIA machines send very weak alternating current through the body, and body tissue resists this current. The Inbody 770 and Tanita 980 divide the human body into five cylinders in order to increase the accuracy of the measure, and both machines deliver currents of 50-1,000 kHz.

Participants were required to stand on the balance scale pare feet, and hold the handles of the machines, following very simple audio structures and visual animation of appropriate position. The measurements take around 15 s, and output is printed.

#### Lean mass and sarcopeania calculation

Total and percent lean mass and TLM/ht^2^ were calculated. ALM is the sum of arm and leg lean mass, and ALM/ht^2^ was also calculated. Sarcopenia is defined as 2 SD below the average of ALM/ht^2^, but it was calculated in the present study as 1 SD below the mean of ALM/ht^2^.

#### Hand grip and knee extensors strength

Dominant hand grip strength was measured using a manual spring dynamometer (Baseline® Smedley Spring Dynamometers, Fabrication enterprises Inc., NY, USA), the handle was adjusted to confortable hand grip size of participant, and participants were asked to squeeze the handle with their maximal force while standing and elbow was fully extended, with consistent encouragements for all participants, and the best of two measures was recorded in kg. In the same way using Baseline dynamometer, isometric contraction for knee extensors strength was measured while seated with a knee angle of 110° using a manual dynamometer. Participants were asked to push at maximal volitional contraction for 5 s, and the better of two measures was recorded in kg.

### Statistical analysis

Data were analyzed using SPSS software (version 22 Chicago, IL, USA). Continuous data were presented as mean ± standard deviation (SD) and median (1^st^ and 3^rd^) percentile for variables according to Gaussian and non-Gaussian distributions. Categorical data were presented as frequencies and percentages (%). All continuous variables were checked for normality using the Kolmogorov-Smirnov test. Non-Gaussian distributions were log transformed. One-sample t-tests and independent Student’s t-tests or the Mann-Whitney U tests were adopted based on Gaussian and non-Gaussian distributions to identify any differences among various characteristics. The degree of agreement between two methods were measured by Cohen’s kappa (*κ*) ≥0.8 were consider good agreement, and an α-level of 0.05 will be used to determine statistical significance.

## Results

Table 1 shows the physical characteristics of young Saudi men. Grip strength was classified based on body mass index (BMI), and the value for 53 participants with BMI below 24 kg/m^2^ was 40.1 ± 6.9 kg, 43.1 ± 7.5 kg in 79 participants with BMI between 24.1 and 28 kg/m^2^. Hand grip strength values was 44.1 kg in 100 participants with BMI greater than 28 kg/m^2^, and the difference was significant when compared with participants with BMI below 24 kg/m^2^ (*P* = 0.006).

**Table 1:** Characteristics of young Saudi men (N = 232)

Parameters	Mean ± SD
Age (years)	27.09 ± 4.18
Height (cm)	171.85 ± 6.05
Weight (kg)	83.30 ± 18.11
Body mass index (kg/m^2^)	28.12 ± 5.48
Waist circumference (cm)	92.13 ± 14.28
Waist-to-hip ratio	0.87 ± 0.06
Knee extensors strength (kg)	78.09 ± 23.95
Grip strength (kg)	42.88 ± 7.61

Table 2 shows the mean value of ALM/ht^2^ plus 1 and 2 SD; ALM/ht^2^ is considered the main sarcopenia index. It should be noted that only two (DXA and Inbody) to four (Tanita) participants had values 2 SD lower than the mean, and then classified as sarcopenic class 2.

**Table 2:** Distribution of appendicular lean mass to height squared (ALM/ht^2^) among young Saudi men (N = 232)

Parameters	DXA *n* (%)	Inbody 770 *n* (%)	Tanita 980 *n* (%)
Mean	8.97 197 (84.9%)	8.16 195 (84.1%)	9.55 173 (74.6%)
1 SD Sarcopenia Class 1	7.74 33 (14.2%)	7.29 35 (15.1%)	8.68 55 (23.7%)
2 SD Sarcopenia Class 2	6.51 2 (0.9%)	6.42 2 (0.9%)	7.45 4 (1.7%)

*DXA* dual-energy X-ray absorptiometry

Table 3 shows the differences between normal and sarcopenic participants defined as 1 SD below the mean of ALM/ht^2^ in anthropometry and muscle strength, whereas Table 4 shows the differences in the sarcopenic indices using the three methods of muscle mass measurement. The differences between DXA and Tanita measurements were significant for all parameters, but the differences between DXA and Inbody measurements were significant only for ALM parameters. Using G*power calculator for sample size determination using the effect size (small) =0.50. β/α ratio =1,sample size for 1^st^ group = 197,sample size for 2^nd^ group = 37,we obtained the actual sample size for this study 234 with actual power achieved =0.91.

**Table 3:** Comparing anthropometric characteristics of normal and sarcopenic (class 1) young Saudi males, using different methods of measures

	Dual-energy X-ray absorptiometry (DXA)			Bioelectrical impedance analysis (BIA)					
	DXA			Inbody 770			Tanita 980		
Variables	Normal	Sarcopenia	*P* value	Normal	Sarcopenia	*P* value	Normal	Sarcopenia	*P* value
Age	27.1 ± 4.2	27.0 ± 4.2	0.89	27.2 ± 4.2	26.6 ± 4.4	0.59	27.2 ± 4.1	26.9 ± 4.4	0.71
Height	172.2 ± 6.1	170.2 ± 5.7	0.09	172.7 ± 5.9	167.6 ± 4.9	**<0.001**	172.2 ± 5.9	171.3 ± 6.5	0.41
Weight	86.7 ± 17.3	64.1 ± 7.4	**<0.001**	86.9 ± 17.2	63.9 ± 7.9	**<0.001**	89.2 ± 16.8	66.1 ± 7.9	**<0.001**
BMI	29.2 ± 5.2	22.1 ± 2.3	**<0.001**	29.1 ± 5.3	22.9 ± 2.8	**<0.001**	30.1 ± 4.9	22.5 ± 2.0	**<0.001**
Knee extensors Strength	80.2 ± 23.5	66.6 ± 23.7	**0.002**	80.4 ± 23.7	66.2 ± 21.8	**<0.001**	79.4 ± 23.9	74.4 ± 24.0	0.17
Grip Strength	43.8 ± 7.5	38.0 ± 6.6	**<0.001**	43.8 ± 7.5	38.0 ± 6.3	**<0.001**	43.8 ± 7.6	40.3 ± 7.2	**0.003**

Data represented Mean ± SD. *P*-value < 0.05 & 0.01 level will be significant

*BMI* body mass index

**Table 4:** Muscle mass indices (mean ± SD) using DXA and BIA

Variables	Dual-energy X-ray absorptiometry (DXA)	Bioelectrical impedance analysis (BIA)	
		Inbody 770	Tanita 980
Total lean mass (kg)	53.75 ± 8.42	53.99 ± 7.20	58.19 ± 7.84**
Lean mass (%)	65.93 ± 8.62	66.37 ± 8.91	71.46 ± 7.02**
TLM/ht^2^ (kg/m^2^)	18.31 ± 2.20	18.33 ± 1.85	19.7 ± 2.14**
Appendicular lean mass (kg)	26.65 ± 4.44	24.12 ± 3.48**	28.26 ± 4.35**
ALM/ht^2^ (kg/m^2^)	8.97 ± 1.23	8.16 ± 0.87**	9.55 ± 1.23**

**P-value < 0.01 compared with DXA

*TLM* total lean mass, *ALM* appendicular lean mass

**Fig. 1:**
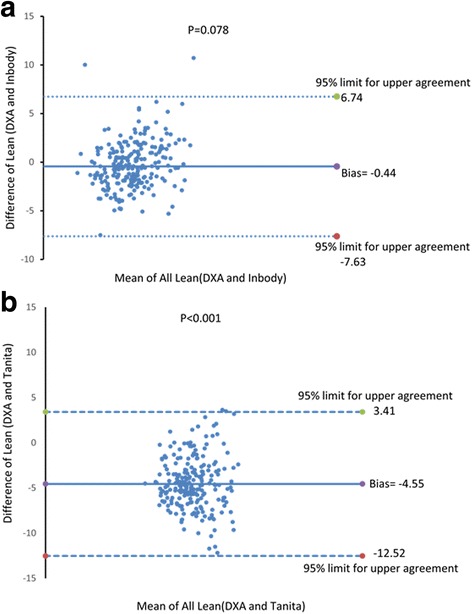
Bland Altman plot for difference between lean values of Inbody and DXA (**a**) and Tanita and DXA (**b**)

The sensitivity and specificity results of Inbody and Tanita measurements for agreement with DXA using kappa values showed that Inbody sensitivity and specificity were 73% and 95.9%, respectively, and the kappa (*P* = 0.80) and *P*-values (*P* < 0.001). On the other hand, Tanita only showed agreement with the DXA kappa value (*P =* 0.61) and *P*-value (*P* < 0.001), with sensitivity and specificity of 54.2% and 98.3%, respectively. Figure 1 shows the Bland-Altman plots for differences between Tanita, Inbody, and DXA lean mass measurements, showing very high bias for Tanita and DXA methods, with significant differences (*P* < 0.001).

## Discussion

The current study aimed to determine the mean reference values for sarcopenia indices among healthy young Saudi men, using DXA and two body composition analyzers. The main outcomes is that among 232 young men, the reference value of ALM/ht^2^, that should be used by future studies on elderlies, was 8.97 kg/m^2^ using DXA, and this represented the majority of participants (84.9%), whereas 14.2% of participants were classified as sarcopenic class 1 (7.74 kg/m^2^), and only 0.9 were classified class 2 (6.51 kg/m^2^). Data showed differences in the mean values of muscle strength and muscle mass, compared with East Asian and American reference values. Interestingly, prior studies reported general reference values for BIA without discriminating between different brands and models, whereas the current study showed significant differences between the most common BIA devices.

One criterion of sarcopenia is hand grip strength, which was determined to be 30 kg as recommended by the EWGSOP using a cohort of 1,030 Italian participants aged between 20 and 102 years [16]. The AWGS recommended a cut-off of 26 kg for men, based on several epidemiological studies in Asia [13]. In a large-scale study that included more than 100,000 adults from 21 countries, ethnicity and geographic regions played a strong role in the diversity of hand grip strength; at 43 kg, grip strength was intermediate for participants from the Middle East, and ranged from 37 to 48 kg among Arabic men aged 35–40 years [17]. Although the current cohort was younger, another study found that the mean value of hand grip strength in North American men in the age group 40–44 years (54.1 kg) was greater than the grip strength in the age group 30–34 years (52.8 kg), and similar to that in the age group 25–29 years (53.9 kg), with no significant differences between groups [18]. Hand grip strength reaches a peak at 35 years, and decreases thereafter [19]. Grip strength was significantly influenced by BMI, and other factors such as age can also affect this relationship, particularly after 30 years of age [8]. Interestingly, sarcopenic men, based on appendicular muscle mass, had lower hand grip strength. Thus, the average hand grip strength for the current cohort was similar to that in previously reported studies among Arabs, but higher than that in Asians and Europeans, and lower than the average levels for American men.

The ALM cut-off was 6.51 kg/m^2^, determined as 2 SD below the mean value in young healthy men (8.97 kg/m^2^) using DXA, which was significantly different when using BIA devices (Table 2). This was lower than the EWGSOP cut-off, which was determined to be either 7.26 kg/m^2^, using a value 2 SD below that of healthy young adults, or 7.25 kg/m^2^, using the 20^th^ percentile of Americans aged 70–79 years [16]. Another study in a Shanghai population determined the cut-off to be 6.66 kg/m^2^ for men using BIA, with a mean value for young men of 7.9 kg/m^2^ [20]. The ALM cut-off was 6.76 kg/m^2^ for young Taiwanese men, using the Tanita BC-418 BIA [6]. The AWGS determined an ALM cut-off at 7.0 kg/m^2^, using either DXA or BIA [13]. There is evidence that a universal cut-off of ALM, ALM/ht^2^, BMI and grip strength are not applicable for some ethnic groups, and there is a considerable difference between the same ethnic groups who live in different geographical locations, and the difference between Asians groups were greater than between Caucasians [21]. Another study found significant differences in sarcopenia prevalence among Chinese population when using Asian compared with Western criteria [22]. Collectively, our results and previous studies suggested the importance of establishing ethnic-specific set of reference value of sarcopenia.

The high specificity and low sensitivity of the Tanita BIA indicated that it can correctly classify individuals with sarcopenia, but may also classify healthy individuals as sarcopenic. The Inbody BIA showed high specificity and high sensitivity, with a high kappa value. Our finding was similar to a study showing that the correlation between DXA and BIA using the Inbody 770 was high (r^2^ = 0.95), and that systematic differences in the Bland-Altman plot were not significant (−0.33 ± 2.02 kg, P = 0.31) [20]. Although a study showed that Inbody 720 tended to overestimate lean mass and underestimate FM compared with DXA [23], good agreements were observed between Inbody 720 and DXA in estimating whole body lean mass (ICC for women = 0.95, ICC for men = 0.96), and BIA underestimated total lean mass by 1.8% [24]. Narrow limits of agreement with small biases were observed for lean mass using Bland-Altman methods, whereas the limits of agreement were wider and appeared to overestimate fat mass and percentage fat mass, which increased with BMI [24]. Another study found that the correlation between Inbody 720 and DXA in measuring FM and FFM in obese women was high (ICC = 0.83 and 0.89, respectively). It is important that the producers of new developed Tainita and Inbody devices improved algorithm using basic information provided by scientific equations and additional information collected from thousands of people worldwide. Thus, the current outcomes used data provided by devices, and differences between Tanita and Inbody could be attributed to the algorithm developed by these devices. Apparently, Inbody models using direct segmental multi-frequency technique are accurate, based on current outcomes and previous studies.

Different Inbody models may lead to different results. For example, Inbody S10 demonstrated systematic overestimation of muscle mass compared to DXA [25], and the agreement of ALM/ht^2^ assessed by Inbody S10 (9.19 ± 1.39 kg/m^2^) and DXA (7.34 ± 1.34 kg/m^2^) was low (ICC = 0.37, *P* < 0.001) [26]. The portable Inbody 230 is acceptable for estimation of FM and FFM, particularly for healthy men, but it is not appropriate to estimate appendicular FFM, and must be reevaluated by the manufacturer before it can be used for the measurement of sarcopenia [27]. Thus, it is important that the current recommendation for use of an Inbody device in sarcopenia analysis be specified as the Inbody 770.

Furthermore, six-minute walk test (6 MW) has been widely used to evaluate functional capacity in sarcopenia studies, and can be used to calculate gait speed. Although 6 MW was not examined in the current study, Alameri et al. [28] examined it among 298 healthy volunteers age between 16 and 50 years in Saudi Arabia, and found that the average 6 MW was 429 ± 47 m for males, and predict equation of the distance was (2.81 * height + 0.79 * age – 28.5). Gait speed as presented by 6 MW distance among Saudis was different compared to other references. For example, reference values of 6 MW among Bangladeshi healthy adults of average age 37.9 ± 8.5 years were 466.7 ± 69.4 m [29]. A study, conducted on 102 Caucasian adults between 20 and 50 years old, found that the mean distance completed during 6 MW was 593 ± 57 m for women and 638 ± 44 m for men, and height, age and gender accounted for 42% of the variation of 6 MW distance [30].

### Strength and limitations

This is the first report of reference values for sarcopenia indices in young Saudi men. This study used the latest and most accurate models of DXA, Tanita, and Inbody devices. Although participants came from different parts of the city of Riyadh and other nearby cities, one of the limitations is that we could not recruit from different cities and rural areas to represent the general population of Saudi Arabia. In a study included several regions in the world including Saudi Arabia, significant variations in hand grip strength were observed among regions, and dietary patterns such as protein intake variation and socioeconomic states partially explain the variation in muscle mass and muscle strength [17]. A recent review showed some regional differences in overweight, obesity, and abdominal obesity among Saudi adolescents, with the highest prevalence in the Eastern Region of Saudi Arabia [31]. It is not evident yet whether such variations include differences in muscle mass and muscle strength patterns. A multi-centered study is needed to accurately represent the diversity of geographical and socioeconomic diversity in Saudi Arabia.

## Conclusion

The current study showed that the cut-off values for sarcopenia indices in Saudi young men were different from previously reported values for other ethnicities and geographic locations. In addition, there were significant differences in the results using different BIA devices; therefore, use of different devices for measurement requires utilizing cut-off reference values that are specific for these devices.

## Abbreviations

ALM: Appendicular lean mass
BIA: Bioelectrical impedance analysis
BMI: Body mass index
DXA: Dual-energy X-ray absorptiometry
TLM: Total lean mass
